# Growth and Molecular Responses of Tomato to Prolonged and Short-Term Heat Exposure

**DOI:** 10.3390/ijms24054456

**Published:** 2023-02-24

**Authors:** Mirta Tokić, Dunja Leljak Levanić, Jutta Ludwig-Müller, Nataša Bauer

**Affiliations:** 1 Department of Molecular Biology, Faculty of Science, University of Zagreb, 10000 Zagreb, Croatia; 2 Faculty of Biology, Technische Universität Dresden, 01062 Dresden, Germany

**Keywords:** ABA, ACC, DREB, HSF, HSP70, HSP90, heat stress induced transcription factors, IAA, NAC, root architecture

## Abstract

Tomatoes are one of the most important vegetables for human consumption. In the Mediterranean’s semi-arid and arid regions, where tomatoes are grown in the field, global average surface temperatures are predicted to increase. We investigated tomato seed germination at elevated temperatures and the impact of two different heat regimes on seedlings and adult plants. Selected exposures to 37 °C and heat waves at 45 °C mirrored frequent summer conditions in areas with a continental climate. Exposure to 37 °C or 45 °C differently affected seedlings’ root development. Both heat stresses inhibited primary root length, while lateral root number was significantly suppressed only after exposure to 37 °C. Heat stress treatments induced significant accumulation of indole-3-acetic acid (IAA) and reduced abscisic acid (ABA) levels in seedlings. As opposed to the heat wave treatment, exposure to 37 °C increased the accumulation of the ethylene precursor 1-aminocyclopropane-1-carboxylic acid (ACC), which may have been involved in the root architecture modification of seedlings. Generally, more drastic phenotypic changes (chlorosis and wilting of leaves and bending of stems) were found in both seedlings and adult plants after the heat wave-like treatment. This was also reflected by proline, malondialdehyde and heat shock protein HSP90 accumulation. The gene expression of heat stress-related transcription factors was perturbed and *DREB1* was shown to be the most consistent heat stress marker.

## 1. Introduction

Temperatures vary geographically and are predicted to rise with global warming, presenting serious threats to agricultural productivity. In the upcoming years, heat stress will become a major abiotic stress factor for many crop species. Not only global temperatures increase, but also the more frequent and severe heat waves will have a considerable impact on ecosystem changes and crop loss worldwide. As sessile organisms, plants are mercilessly exposed to environmental conditions. The stress response in plants is a complex trait regulated by many factors and can decrease plant performance. Mild stress retards plant growth, activates defense mechanisms and may change growth and developmental patterns. Intense stress, however, stops growth, causes the accumulation of harmful metabolites and may even cause plant death. A rich repertoire of flexible mechanisms that control gene expression enables plants to exhibit a quick response to external signals and to readily adapt to a plethora of environmental conditions [1].

Tomato (*Solanum lycopersicum*) is a fruit vegetable crop from the Solanaceae family cultivated worldwide, and as such, it is frequently exposed to extreme temperature fluctuations. After the juvenile stage, in adult plants vegetative and reproductive development occur simultaneously. Tomatoes originate in sub-tropical areas but can be grown in greenhouses throughout the whole year if the temperature is appropriately regulated. Temperature significantly impacts tomato growth and development. The optimum temperature for growth and a high fruit yield ranges from 20 °C to 25 °C during the day and from 18 °C to 25 °C during the night [2]. The effects of varying temperatures on tomato cultivation have been meticulously described [2,3].

Heat stress is defined as a condition where the ambient temperature is between 10 °C and 15 °C higher than the optimum temperature range for plant cultivation. Whereas the rise in temperature could stimulate growth, heat stress causes negative effects on plant morphology, physiology and biochemistry [4]. Heat stress can significantly affect cellular homeostasis, including changes in photosynthesis, protein misfolding and/or aggregation, the accumulation of reactive oxygen species and cell membrane damage [5]. The plant growth regulators play a role in stress response and integrate environmental stimuli and endogenous signals to regulate plant growth and development. Heat stress, especially under water deficit, elicits a rapid and transient increase in endogenous abscisic acid (ABA), which then suppresses growth and coordinate adaptation to stressful conditions. Auxin (especially its most prominent form, indole-3-acetic acid, IAA) is significantly increased in seedlings grown under heat stress and influences thermomorphogenesis by inducing stem elongation and leaf hyponasty, while the role of ethylene in plant response to heat stress varies, and additional studies are needed to clarify its role in heat stress responses [6,7,8,9]. High temperatures induce the synthesis of heat shock proteins (HSPs) that are molecular chaperones and play a vital role in protecting the stability and functional conformation of cellular proteins. HSPs recognize and bind exposed hydrophobic regions of misfolded proteins and prevent protein aggregation. In addition, HSP70 and HSP90 negatively regulate heat stress transcription factors (HSFs). Under normal growth conditions the inactive state of HSFs is maintained by the HSP70/HSP90 complex, while under heat stress partially denatured cellular proteins compete with HSFs for HSP70/HSP90 binding. As a consequence, HSFs are released from the complex and translocated to the nucleus [10,11,12]. They bind to heat shock elements in target gene promoters and regulate their transcription [10,13].

Here, we aimed to evaluate the impact of different heat regimes, partially mimicking global warming, on tomato growth and development. We determined the maximum permissive germination temperature and the maximum exposure time at 45 °C that still allowed tomato seedlings to survive. We also analyzed changes in physiological parameters, including proline; malondialdehyde (MDA); ABA; IAA; and 1-aminocyclopropane-1-carboxylic acid (ACC), the immediate precursor of ethylene, as well as molecular markers, HSP70 and HSP90 protein accumulation and heat stress-related gene expression levels, upon prolonged exposure of tomato seedlings and adult plants to a moderately elevated temperature or a short-term heat wave-like exposure. Our results indicate important physiological and molecular differences between the two stress regimes.

## 2. Results

### 2.1. Tomato Germination and Plant Morphology following Heat Stress

**Figure 1:**
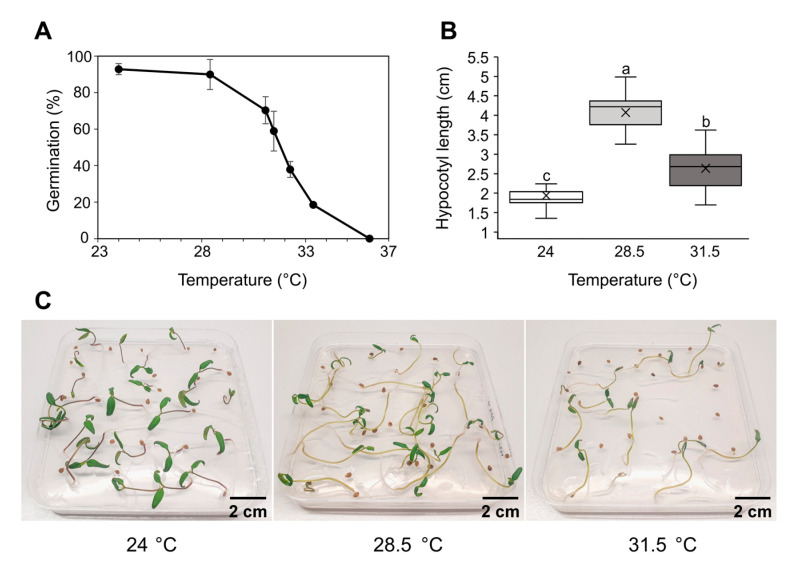
Effect of elevated temperatures on tomato seed germination and seedling development. Following stratification, tomato seeds were exposed for 8 days to a set of rising temperatures after which the germination rate was determined (**A**), hypocotyl lengths were measured (**B**) and seedlings’ phenotype was documented (**C**). Data in (**A**) represent the average of three biological replicates, each consisting of 25–30 seeds. For (**B**), data are represented as boxes indicating the lower and upper quartile, while the means and medians of 20 seedlings are denoted with a horizontal line and a cross in the box, respectively. Different letters indicate a significant difference at *p* < 0.05 (Tukey’s test). The scale bar is 2 cm.

To determine the effect of elevated temperatures on tomato seed germination, stratified seeds were incubated for 8 days at a constant temperature ranging from 24 °C to 37 °C. Germination was completely blocked at 36 °C (Figure 1A). Higher temperatures (≥28 °C) correlated with a reduced germination rate and influenced seedling development. Cotyledon size was reduced at temperatures higher than 24 °C, while hypocotyl length increased (Figure 1B,C). The average hypocotyl length of 8-day-old seedlings germinated at 28.5 °C and 31.5 °C was 4.1 cm and 2.6 cm, respectively, representing a significant difference compared to the length of 1.9 cm in seedlings germinated at 24 °C.

**Figure 2:**
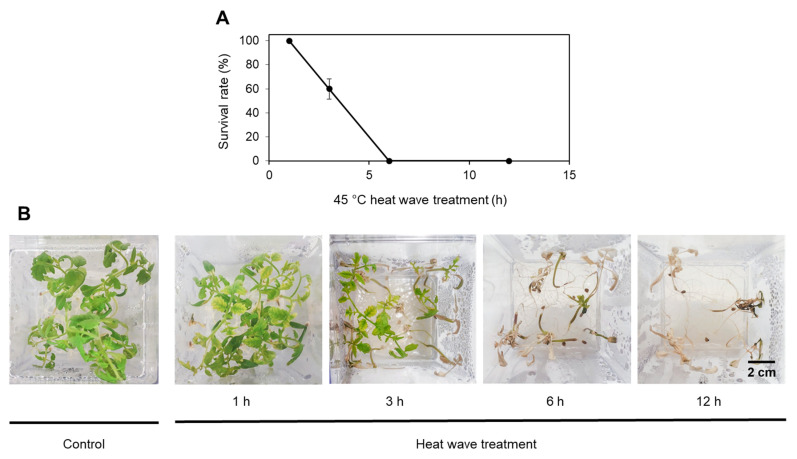
Heat stress tolerance in tomato seedlings. Twelve-day-old seedlings were exposed to 45 °C for 1, 3, 6 and 12 h. Control plants were kept at 24 °C. Survival rates (**A**) were estimated following a 7-day recovery period (Figure S1A). Green seedlings still producing new leaves were scored as survived. Pictures were taken after the 7-day recovery (**B**). Each biological replicate consisted of 15 seedlings. Data represent the mean of 3 replicates with standard deviations denoted by vertical bars. The scale bar is 2 cm.

Further, 12-day-old tomato seedlings germinated at 24 °C were exposed to 37 °C or 45 °C, and the survival rate was determined after a 7-day-long recovery (Figure 2 and Figure S1A). Whereas 24 h of exposure to 37 °C and a 1-hour-long heat wave treatment did not compromise seedling survival rate, a 3-hour heat wave exposure at 45 °C caused cotyledon and leaf bleaching and chlorosis. Exposure to 45 °C for 6 or 12 h resulted in seedling desiccation and an inability to recover for further growth (Figure 2). Therefore, the 3-hour-long 45 °C heat wave treatment was used in subsequent experiments on seedlings.

Both heat stress treatments, the continuous exposure to the 37 °C and the 3-hour-long heat wave at 45 °C had a significant impact on seedling growth and development. The average biomass accumulation of tomato seedlings exposed to 37 °C (39.3 mg) and 45 °C (58.4 mg) showed an obvious reduction compared to the control (70.8 mg).

**Figure 3:**
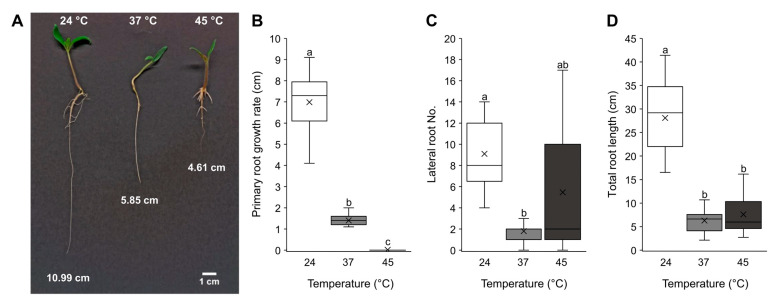
Effects of heat stress on seedling root development. Seven-day-old seedlings were exposed to 37 °C or to heat wave at 45 °C for 3 h and further cultured at 24 °C (Figure S1B). Control plants were kept at 24 °C. The seedlings were analyzed and photographed (**A**) 5 days after the start of heat treatments. Primary root growth rate (**B**), lateral root number (**C**) and total root length (**D**) were measured. Data are represented as boxes that indicate the lower and upper quartile while means and medians of 15 seedlings are denoted with a horizontal line and a cross in the box, respectively. Whiskers represent the highest and lowest scores in the data set. Different letters indicate a significant difference at *p* < 0.05 (Tukey’s test). The scale bar is 1 cm.

Heat treatments disturbed root development by significantly inhibiting primary root growth and disturbing lateral root development (Figure 3). At 37 °C, lateral root formation was extremely reduced, and primary root growth was inhibited. By contrast, the 45 °C treatment compromised lateral root initiation less but arrested primary root growth (Figure 3A–C). Both treatments significantly reduced total root length (Figure 3D).

Finally, 11-week-old adult tomato plants were heat treated at 37 °C for 24 h or at 45 °C for 5 h (Figure S1D). After the 5 h treatment, plants exhibited symptoms equivalent to seedlings treated at 45 °C for 3 h (wilting leaves and bending stems). Plants treated at 37 °C had mildly bent leaves and showed no apparent differences compared to control plants. Although both treated plant groups were revived after 7 days at control conditions, the wilted yellow leaves caused by the exposure to 45 °C never recovered.

### 2.2. Heat Stress Effects on IAA, ACC and ABA Accumulation

**Figure 4:**
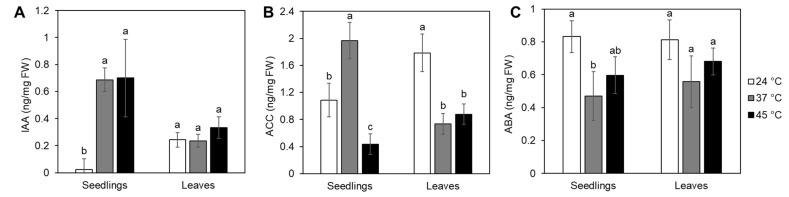
Phytohormone levels in tomato seedlings and adult plant leaves. Seedlings or adult plants were treated either continuously at 37 °C or 45 °C followed by recovery. Control plants were kept at 24 °C (Figure S1C,D). (**A**) Indol-3-acetic acid (IAA), (**B**) 1-aminocyclopropane-1-carboxylic acid (ACC) and (**C**) abscisic acid (ABA) were measured from whole seedlings or leaves harvested 24 h from the start of the treatments and expressed per fresh weight (FW). Each biological replicate consisted of 15 seedlings or leaves pooled from 6 adult plants. Data represent the average of 3 replicates with standard deviations denoted by vertical bars. Different letters indicate a significant difference between control and heat treatments at *p* < 0.05 (Tukey’s test) for each tissue type separately.

Due to changes in root growth and development provoked by heat stress, we examined the effects of prolonged heat exposure and heat wave on ABA, ACC (the precursor of ethylene) and IAA accumulation. These plant hormones were selected based on their well-known roles in stress responses and regulation of root development. Both types of heat stress treatments significantly induced IAA accumulation in seedlings (Figure 4A), while the heat wave caused a slight but not significant elevation of IAA in the leaf tissue of adult plants (Figure 4A). The different heat treatments had opposite effects on ACC accumulation in seedlings. At 37 °C, ACC was induced, while it decreased at 45 °C (Figure 4B). Both heat treatments significantly reduced ACC levels in adult plant leaves (Figure 4B). ABA content decreased in both heat-treated seedlings and adult plant leaves Figure 4C), although significance was only observed in seedlings exposed to 37 °C.

### 2.3. Heat Stress Effects on Proline and Malondialdehyde Accumulation

**Figure 5:**
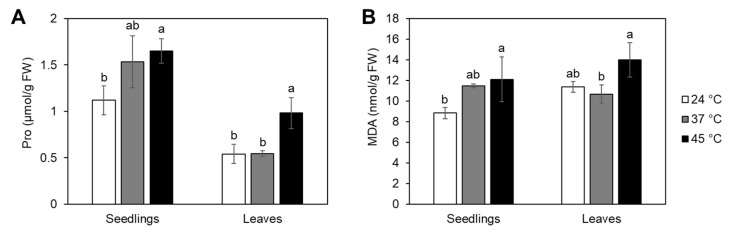
Proline (Pro) and malondialdehyde (MDA) content in tomato seedlings and adult plant leaves under heat stress. Seedlings or adult plants were either treated continuously at 37 °C or at 45 °C followed by recovery. Control plants were kept at 24 °C (Figure S1C,D). Pro (**A**) and MDA (**B**) contents were measured from whole seedlings or leaves harvested 24 h from the start of the treatments and expressed per fresh weight (FW). Each biological replicate consisted of 15 seedlings or leaves pooled from 6 adult plants. Data represent the average of 3 replicates with standard deviations denoted by vertical bars. Different letters indicate a significant difference between control and heat treatments at *p* < 0.05 (Tukey’s test) for each tissue type separately.

To estimate the severity of heat treatments on tomato seedlings and adult plants, proline and MDA accumulation were measured. Seedlings and adult plants were heat treated at 37 °C for 24 h or 45 °C for 3 or 5 h, respectively. In seedlings, proline and MDA levels were enhanced upon exposure to both heat regimes, significantly increasing only after the heat wave treatment (Figure 5). Adult plants exposed to 37 °C showed no change in either proline or MDA levels, while the heat wave treatment significantly induced proline accumulation (Figure 5).

### 2.4. Heat Stress Responsive Transcription Factor Expression Quantification

**Figure 6:**
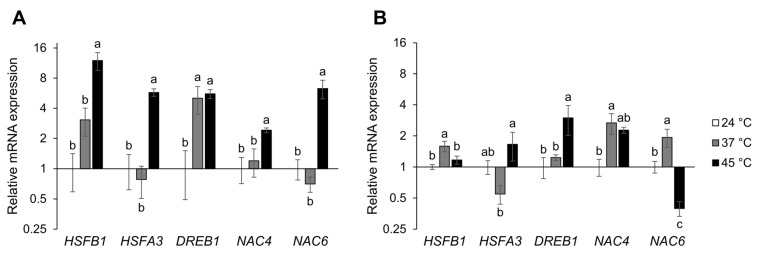
Gene expression analysis of tomato heat stress-related transcription factors. Seedlings (**A**) or adult plants (**B**) were either treated continuously at 37 °C or short exposed to 45 °C followed by recovery. Control plants were kept at 24 °C (Figure S1C,D). RNA was isolated from seedlings or young leaves harvested 24 h after the start of the treatment and expression of *HSFB1*, *HSFA3*, *DREB1*, *NAC4* and *NAC6* genes was quantified. Each biological replicate consisted of 15 seedlings or leaves pooled from 6 adult plants. The data are expressed as ΔΔCq values on a log2 scale normalized to tomato genes *ACT* (for leaves), or *EFI*-α (for seedlings) and control (24 °C). The average of 3 biological replicates with standard deviations denoted by vertical bars are presented. Different letters indicate significant differences at *p* < 0.05 (Tukey’s test).

To analyze the response of heat-treated tomato, relative transcript abundance of heat stress-related transcription factors *HSFB1*, *HSFA3*, *DREB1*, *NAC4* and *NAC6* was measured by qPCR. In seedlings, the heat wave-like treatment significantly induced the expression of all tested heat stress-related transcription factors, while only *DREB1* expression was significantly induced at 37 °C (Figure 6A). In adult plant leaves at 37 °C, *HSFB1*, *NAC4* and *NAC6* expression were significantly induced. Exposure to 45 °C induced *HSFA3* and *DREB1* but reduced *NAC6* expression (Figure 6B). The exposure to 37 °C slightly reduced the expression of *HSFA3* in both seedlings and adult plants.

### 2.5. HSP70 and HSP90 Accumulation after Heat Stress Exposure

**Figure 7:**
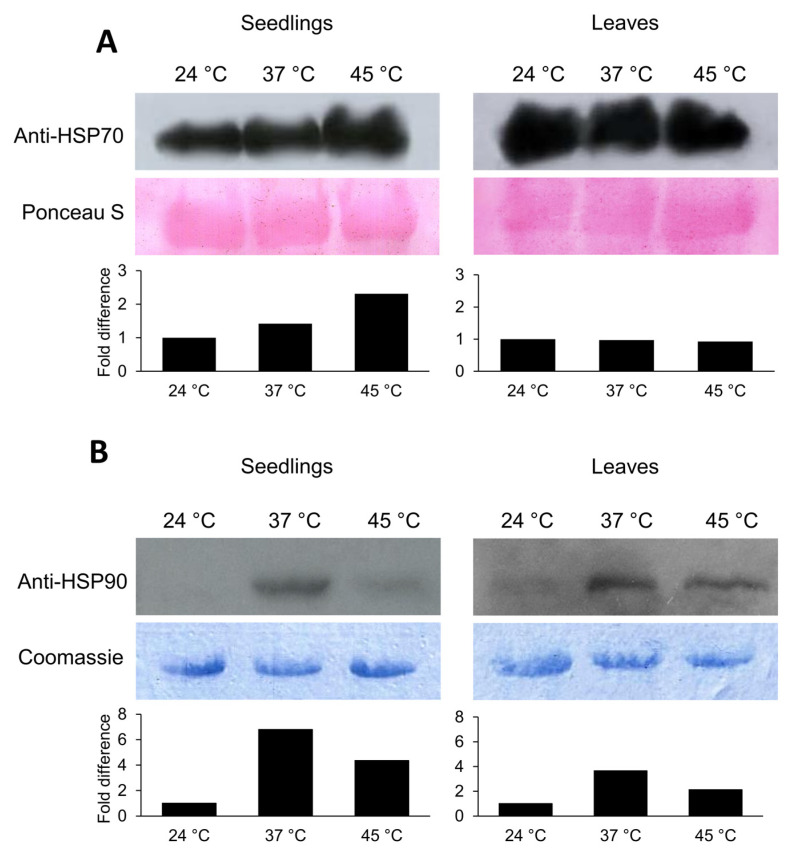
Heat stress effects on HSP70 and HSP90 protein accumulation in tomato seedlings and adult plant leaves. Seedlings or adult plants were either treated continuously at 37 °C or at 45 °C followed by recovery. Control plants were kept at 24 °C (Figure S1C,D). Proteins were isolated from seedlings or young leaves harvested 24 h after the beginning of the treatment. Each biological replicate consisted of 15 seedlings or leaves pooled from 6 adult plants. Immunoassay signals of HSP70 (**A**) and HSP90 (**B**) were normalized to the total protein intensities on nitrocellulose membrane after staining with Ponceau S or PVDF membrane after staining with Coomassie Brilliant Blue.

HSPs are molecular chaperones assisting protein stabilization and refolding under heat stress. To further investigate the heat stress effects on tomato, immunodetection of HSP70 and HSP90 was performed. In contrast to HSP90, which scarcely accumulated in control conditions, large amounts of HSP70 were present in seedlings and adult plant leaves (Figure 7). Heat treatments induced the accumulation of HSP70 and HSP90 proteins in seedlings but did not change HSP70 accumulation in adult plant leaves. In seedlings, HSP70 more readily accumulated at 45 °C, while HSP90 accumulation was more obvious at 37 °C (Figure 7).

## 3. Discussion

Planet Earth is witnessing significant climate changes characterized by a gradual increase in environmental temperature and pronounced heat fluctuations during which the temperatures can exceed 45 °C. Though plants have evolved various mechanisms to overcome changing environmental conditions [14], increasing temperatures have adverse effects on plant morphology, physiology and biochemistry, affect biodiversity, reduce crop yields and impact the quality of agriculturally important species [15]. High temperature inhibits the vegetative growth of tomatoes [16,17], causes flower drop, reduces fruit set [12] and negatively impacts fruit ripening [2,3,18,19]. The genetic and molecular mechanisms in tomato plants underlying heat stress have been recently reviewed [16,20], but there is still a large gap in understanding the complex network of processes involved in the tomato heat stress response.

Many experiments have focused on the fruit development of tomato under heat stress [21,22]. Seedling and vegetative growth, which occur in parallel with reproduction, significantly contribute to the performance of the crop. Seed germination and seedling growth are indeed the most vulnerable stages in a plant’s life cycle. Light and soil temperature are key environmental factors affecting seed germination [23,24]. We exposed tomato seeds, seedlings and adult plants to elevated temperatures and heat wave-like treatments and monitored plant morphology as well as different biochemical and molecular parameters. We demonstrated that the most suitable temperatures for tomato germination ranged between 24 °C and 28 °C (Figure 1), while germination rates and seedling vigor significantly declined at temperatures higher than 28.5 °C. Only 50% of tomato seeds germinated at 31.5 °C, while a total lack of germination occurred at 36 °C. The results we obtained are in accordance with previous studies [23,25], where the highest possible temperature for tomato germination is 34 °C. Tomato seedlings developed at 28.5 °C and 31.5 °C significantly elongated hypocotyls. This is a well-known phenomenon caused by IAA abundance and signaling by which young seedlings move far from the heat-absorbing soil to reach a better environment for growth and development with lower temperatures [26,27].

We further investigated the impact of heat stress on tomato seedling growth and root morphology (Figure 3). Either a prolonged exposure to 37 °C, a temperature mimicking the summer conditions in areas with a continental climate, or a short-term exposure to 45 °C, simulating a heat wave, were applied. The prolonged exposure reduced primary root growth and obstructed lateral root initiation. Exposure to 45 °C, however, blocked primary root growth completely, but seedlings were still able to develop lateral roots. The root system architecture is a major determinant of agronomic productivity and is influenced by changing environmental conditions [28]. The availability of plant hormones and their crosstalk in response to environmental stimuli play a major role in the root system’s development [29]. Therefore, we measured IAA, ABA and ACC (the immediate ethylene precursor) content in heat-treated tomatoes (Figure 4). In accordance with previously reported results [9], both heat stress regimes used in this study induced significant accumulation of IAA in seedlings. It has long been known that auxin positively regulates lateral root formation in most plant species [30,31], although this was not the case here. Despite the significantly increased concentration of IAA, exposure to 37 °C, but not to 45 °C, reduced the tomato seedlings’ capacity to develop lateral roots. The likely cause for this phenomenon may therefore be attributed to the accumulation dynamics of ACC. The twofold increase in ACC levels at 37 °C likely suppressed lateral root induction, which fits the previous description of ACC’s role in tomato [32]. The authors reported enhanced lateral root formation in ethylene-insensitive mutants and inhibited lateral root development when ACC was applied. However, examinations of heat stress effects on ethylene synthesis in different plant species and tissues showed adverse responses [9]. Heat-generated reactive oxygen species [33] indirectly induce ethylene synthesis, which, in turn, participates in stress alleviation [34,35], while excess ethylene production under severe stress suppresses growth and induces senescence [34]. After production, ACC can also be conjugated with malonate, glutamate or jasmonic acid to produce ACC conjugates that are temporarily unavailable for ethylene production [36,37]. Thus, the low amounts of free ACC observed and subsequent retained ability of lateral root initiation under the heat wave treatment may be the result of ACC conjugate production.

Furthermore, the plant hormone ABA has a role in the modulation of root architecture and can induce both root elongation and lateral root initiation [38]. The heat treatments applied here reduced ABA in tomato seedlings. In addition to elevated ACC and IAA levels, lower ABA likely contributed to inhibited tomato root growth and lateral root initiation in seedlings at 37 °C. Elevated ABA is considered a good stress marker, and recent work in grapes [39] has associated ABA as a suitable marker for drought but not heat stress. Previous studies on 5-week-old tomato plants showed free ABA levels to increase during heat exposure to 35 °C and 45 °C compared to control plants cultivated at 25 °C [40]. In that experiment, ABA levels were the highest (1.5-fold) 12 h after heat exposure and continued to decrease for 48 h. Compared to [40], the different tomato response observed here could have been the result of the different experimental setups, including different cultivar types and plant ages used, as well as a difference in applied temperature regimes.

Proline and MDA are considered reliable indicators of environmental stress severity in tomato [41,42]. To further assess stress severity in heat-treated seedlings and adult plants, proline and MDA accumulations were measured (Figure 5). Proline acts as an osmolyte or molecular chaperone [43]. Proline has been shown to negatively affect ABA and ethylene biosynthesis in Arabidopsis seedlings in particular during heat stress [44]. As a result, the observed significant proline induction could be involved in the reduction of ABA and ACC upon the heat wave treatment in tomato. MDA is a byproduct of lipid peroxidation under environmental stress. Although MDA was shown to function as a protector [45], excess amounts often point to impaired cellular function. Compared to the prolonged treatment at 37 °C, higher proline and MDA levels at 45 °C indicated stronger stress severity in both seedlings and adult plants.

Stress perception, signaling and response are highly regulated at the transcriptional level and lead to the accumulation of different stress-responsive factors. These processes are governed by stress-related transcription factors such as those from the HSF, DREB and NAC families. Plant-specific NAC transcription factors are involved in a multitude of biological processes, from plant growth and development to stress response [46,47]. In tomato, NAC4 and NAC6 are specifically known to be stress-responsive [46,48,49]. Onset, early response and long-term acclimation to heat stress are controlled and regulated by HSFs [50]. In the tomato genome, 26 HSFs are present [51], among which HSFA1, HSFA2 and HSFB1 have been described as master regulators of the heat stress response [52,53,54,55]. Moreover, HSFA3 was shown to be important for heat stress memory [56]. DREB transcription factors play vital roles during heat and water stress responses by influencing the transcription of, among others, HSF genes [57,58]. Since neither ABA nor proline or MDA could be considered reliable stress markers in plants exposed to 37 °C, heat stress-related gene expression and HSP protein abundance were analyzed to further investigate the tomato heat stress response (Figure 6 and Figure 7). In line with proline and MDA accumulation in tomato seedlings grown at 45 °C, the expression of all tested genes (*HSFB1*, *HSFA3*, *DREB1*, *NAC4* and *NAC6*), as well as the accumulation of HSP70 and HSP90 proteins, were significantly induced. Only *DREB1* expression was significantly induced also at 37 °C, indicating its possible role as a master regulator of heat perception and its use as an early and sensitive heat stress marker in tomato seedlings. Although *HSFA3* was previously shown to be heat-responsive [59], an induction was only observed in seedlings exposed to the heat wave treatment, possibly triggering physiological memory formation.

Significant gene expression changes were observed in the tomato leaf tissue. The induction of *HSFB1*, *NAC4* and *NAC6* levels at 37 °C points to a possible role of these genes in the heat response. In contrast, *NAC6* was strongly reduced at 45 °C, comparable to results reported by [49] who found a strong reduction of *NAC6* expression in the leaves of 35-day-old tomato plants exposed to 40 °C. The results therefore indicate a complex, stress-specific *NAC6* gene expression pattern. *DREB1* showed elevated transcript amounts in heat-treated leaves at 45 °C, highlighting the gene yet again as a possible heat stress marker.

Lastly, heat stress exposures caused a notable accumulation of HSP70 and HSP90 in tomato seedlings. HSP70 accumulated more prominently at 45 °C, while HSP90 accumulated more prominently at 37 °C. Accordingly, and in contrast to HSP90, the high level of HSP70 correlated with induced *HSFB1* and *HSFA3* expression in seedlings. HSPs release HSFs in response to heat stress which regulate target gene transcription in the nucleus, among which are HSP genes [10,13]. Consequently, HSPs and HSFs are in constant interaction and interdependence jointly relieving the negative effects of heat stress. Other clients for HSP90 include the auxin receptors from the TIR1 family and might therefore be directly connected to auxin induced growth during heat stress, since a positive effect on Arabidopsis seedling growth was reported through stabilization of the receptor by HSP90 [60]. Whether HSP90 might exert a similar function in tomato has yet to be investigated.

**Figure 8:**
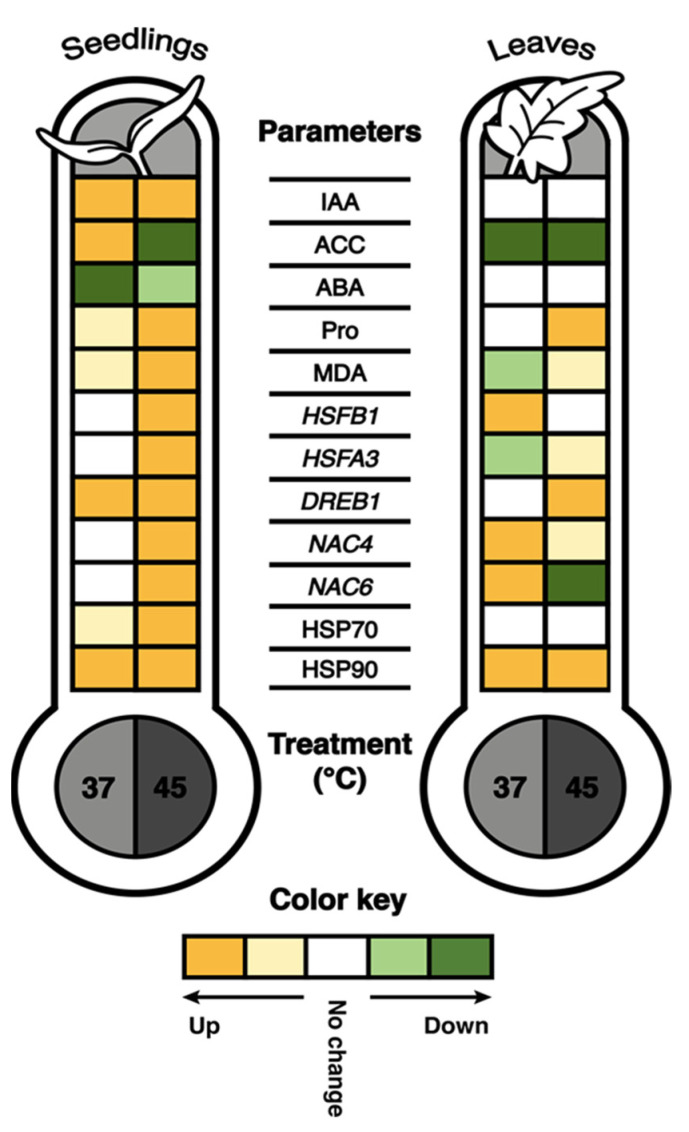
Integrated summary of heat stress effects on all parameters in tomato seedlings and adult plant leaves determined. Prolonged exposure to 37 °C and a short-term treatment at 45 °C were applied. Measured and quantified were phytohormones indol-3-acetic acid (IAA), 1-aminocyclopropane-1-carboxylic acid (ACC) and abscisic acid (ABA); heat stress markers proline (Pro) and malondialdehyde (MDA); heat stress-related transcription factors *HSFB1*, *HSFA3*, *DREB1*, *NAC4* and *NAC6* expression; and heat shock proteins HSP70 and HSP90 accumulation. Colors indicate changes under heat stress compared to corresponding controls (plants kept at 24 °C). The significant up- or downregulation at *p* < 0.05 (Tukey’s test) or fold change ≥ 2 (for HSP70 and HSP90) are represented by intense color. Lighter colors indicate a trend of up- or downregulation, while white displays no change.

In conclusion, our work has shown a complex response pattern of tomatoes to heat stress. The global climate change and expected temperature rise in the future will assuredly impede tomato seed germination and plant development, and consequently affect not only commercially important fruit yield but also tomato biodiversity and its geographical distribution [61]. Our results indicate that the tomato heat stress response is a complex, developmental stage- and heat stress type-dependent process. The heat waves, which are expected to appear more frequently by climate change, caused more pronounced deviations than prolonged exposure to 37 °C. In Figure 8, the results of all biochemical and molecular parameters are summarized. Both types of heat stress caused an increase in proline, IAA, HSP70 and HSP90 proteins and *HSFB1*, *DREB1* and *NAC4* gene expression, but reduced ABA levels in seedlings. Expectedly, adult plants were more resilient to heat stress. Both treatments significantly reduced ACC levels in adult plants. At 45 °C, proline, HSP90 and *DREB1* increased outstandingly and can therefore serve in the future examination as stress markers for the evaluation of heat stress effects on tomato or be considered targets during the generation of thermotolerant tomato plants either by bioengineering or by molecular breeding. Finally, we would like to emphasize the negative effect of heat stress on root development and growth in seedlings, which appears to be connected to ACC production in tomato and needs to be addressed in more details in the future.

## 4. Materials and Methods

### 4.1. Plant Material and Cultivation

Tomato (*Solanum lycopersicum* L.) cultivar Ailsa Craig (Seed Megastore, Nuneaton, UK) was chosen based on its heat tolerance known from previous studies and its sequenced genome [62,63,64]. For all in vitro assays, seeds were surface sterilized by soaking in 70% EtOH for 1 min and then in 2.5% NaOCl and 0.02% Triton X-100 for 30 min. After a fivefold rinsing step with sterile distilled water, seeds were sown on Murashige and Skoog (MS) medium supplemented with 2% sucrose and 1% agar. Plated seeds were stratified at 4 °C for two days before transferring them to growth chambers in a light/dark cycle of 16/8 h (90–100 µmol/m^2^ s) and 24 °C. Regarding adult plant stress treatments, plants were grown in plastic pots containing steam sterilized commercial soil (Einheitserde Classic Pikiererde CL P, Gebrüder Patzer GmbH & Co. KG, Sinntal, Germany), in a greenhouse at 24 °C, under natural illumination conditions with a photoperiod of 13–15 h (April to June) and a relative humidity of 60%. The 11-week-old plants were exposed to heat treatments.

### 4.2. Heat Stress Procedure

The effect of elevated temperatures on tomato seed germination was examined at different heat regimes, ranging from 24 °C to 37 °C. Seeds (25–30 seeds per 120 × 120 mm Petri dish) were exposed to continuous temperatures for 8 days in a light/dark cycle of 16/8 h. Radicle emergence was taken as a criterion for germination. The germination percentage was calculated from the ratio of germinated and total seeds. On the last day of the experiment, germinated seedlings were photographed, and hypocotyl lengths measured.

The survival rate of 12-day-old seedlings was evaluated by exposing seedlings to 37 °C for 24 h or 45 °C for 1, 3, 6 and 12 h. The percentage of surviving seedlings was estimated after a 7-day recovery period at 24 °C (Figure S1A). Seedlings with continued epicotyl elongation and newly developed true leaves were scored as viable.

All molecular and physiological analyses were performed on 12-day-old seedlings germinated at 24 °C on MS medium in Magenta vessels (Sigma-Aldrich) and on 11-week-old adult plants cultivated in long day conditions. Seedlings and adult plants were exposed to 37 °C continuously for 24 h or to a heat wave treatment at 45 °C, for 3 h or 5 h, respectively (Figure S1). Both heat treatments were set up at roughly 10 a.m. After the heat wave treatment, seedlings and adult plants were cultivated at 24 °C until sampling. Whole seedlings (15 per one biological replicate) or young leaves pooled from 6 adult plants (third to fifth leaf from the top of the stem) were sampled 24 h after the beginning of the heat stress treatments. The samples were frozen in liquid nitrogen and stored at −80 °C.

During all heat treatments, temperatures were monitored and recorded by a data logger Testo 174H (Testo GmbH & Co., Lenzkirch, Germany).

### 4.3. Plant Growth Measurements

To assess the effect of heat treatments on root development, seeds were sown in square Petri dishes (120 × 120 mm), stratified at 4 °C for 2 days and cultured vertically under control conditions for 7 days. Seedlings were either exposed to 45 °C for 3 h and then returned to control conditions until analysis or continuously cultivated at 37 °C. Control seedlings were continuously cultivated at 24 °C (Figure S1). All groups were analyzed simultaneously after 5 days from the start of the treatment when the root tips reached the bottom of the dish in control conditions. At this time point, the plates were photographed, and root growth was assessed. Primary root length, lateral root number and total root length per seedling were measured. Primary root growth rate was expressed as the difference between final primary root length and the root length at the beginning of heat treatment. The average fresh mass per seedling was calculated by weighing 10 whole seedlings at the end of the experiment for each treatment and control. Hypocotyl and root lengths were measured using ImageJ software (NIH, Bethesda, MD, USA).

### 4.4. Phytohormones and Stress Parameters

#### 4.4.1. Phytohormone Content

Endogenous IAA, ABA and ACC were quantified by gas chromatography–mass spectrometry (GC–MS) according to adapted protocols originally described in [65,66,67]. Briefly, 100 ng of labelled standards of ^13^C_6_-IAA (Cambridge Isotope Laboratories, Andover, MA, USA), 200 ng of ^2^H_6_-ABA (Cambridge Isotope Laboratories, Andover, MA, USA) and 100 ng of ^2^H_4_-ACC (Euriso-top GmbH, Saarbrücken, Germany) were added directly to 100 mg of frozen homogenized plant tissue (whole seedlings or leaves). The downstream process and combined derivatization proceeded as described by [66]. After adding anhydrous sodium sulfate and a brief centrifugation at 16,000 *g* for 1 min, solutions were transferred to GC vials and evaporated to dryness in a stream of nitrogen. Following evaporation, an additional step of derivatization was added [68]. Methanol and trimethylsilyl diazomethane (TMSD; diluted 1:100 in diethyl ether) were added in a 1:1 ratio to the dried samples and incubated at room temperature before repeating the evaporation step. In the final step of sample preparation, the dried samples were dissolved in 50 μL ethyl acetate for analysis performed by GC–MS (Varian Saturn 2100T, 3800 GC and 8400 Autosampler). Hormone levels were measured by increasing the temperature from 70 to 280 °C at a rate of 20 °C/min. Three biological (15 seedlings or leaves pooled from 6 adult plants per replicate) and three technical replicates were analyzed per treatment or control. Phytohormone content was determined using the principles of isotope dilution [69] from diagnostic ion ratios of endogenous and labelled hormones at a *m*/*z* of 190/194, 130/136 and 141/145 for ABA, IAA and ACC, respectively.

#### 4.4.2. Proline and Malondialdehyde Content

Fifty mg of frozen homogenized tissue (whole seedlings or leaves) was extracted with 1 mL 70% EtOH and centrifuged at 10,000 *g* and 4 °C for 10 min. Supernatants were used for proline [70] and MDA content determination [71,72]. Three biological (15 seedlings or leaves pooled from 6 adult plants per replicate) and two technical replicates were analyzed per treatment or control sample.

### 4.5. RNA Isolation and Gene Expression Analysis

RNA was isolated from 50 mg of frozen homogenized tissue (whole seedlings or leaves) using the MagMAx Plant RNA Isolation Kit (Thermo Scientific) according to the manufacturer’s instructions. After elution, RNA was quantified by NanoDropTM 1000 Spectrophotometer (Thermo Scientific). Then, cDNA was synthesized from 1 µg of isolated RNA using 200 U of RevertAid H Minus Reverse Transcriptase and 2.5 µM Oligo(dT)18 primer (Thermo Scientific). Quantitative real-time PCR (qPCR) was performed on the MIC platform (Bio Molecular Systems). The reactions included 1× GoTaq^®^ qPCR Master Mix reagent (Promega), 200 nM of forward and reverse primers (Table S1) and 20 ng cDNA in a total reaction volume of 10 µL. The run profile of the PCR reaction was as follows: 95 °C for 5 min, followed by 40 cycles of 95 °C for 5 s and 60 °C for 10 s. In addition, melting curves were generated to check for specific amplification by increasing the temperature from 55 °C to 95 °C at 0.5 °C/s. Relative expression of heat stress-related genes *DREB1*, *HSFA3*, *HSFB1*, *NAC4* and *NAC6* was calculated by the ΔΔCq method [73,74] using *ACT* [75] and *EFI*-α [76] genes as endogenous controls. Three biological (each consisting of 15 whole seedlings or leaves pooled from 6 adult plants) and two technical replicates were analyzed per treatment and control. Genes, accession numbers and primer sequences are from [48,49,51,75,76,77] and listed in Table S1.

### 4.6. Heat Shock Protein Detection

Soluble proteins were extracted from 150 mg frozen homogenized tissue (whole seedlings or leaves) in 0.5 mL of protein extraction buffer (92.5 mM TRIS-HCl, 500 mM sucrose, 6.48 mM DTT, pH 7.6; [78]). Protein concentrations were determined using the Bradford reagent [79]. Proteins (25 μg per lane) were separated on 12%-SDS-polyacrylamide gels and transferred to a PVDF or nitrocellulose membrane. Membranes were blocked in 2% (*w/v*) non-fat dry milk in 1× tris-buffered saline buffer (TBS) overnight at 4 °C. Primary antibodies, anti-HSP90-1 (Agrisera AS08346) or anti-HSP70 (Agrisera AS08371) diluted 1:3000 in 1× TBS, secondary antibody (Anti-Rabbit IgG HRP goat antibody, EMD Millipore diluted 1:50,000), and Immobilon^®^ Forte Western HRP substrate, (Millipore) were used for HSP90 and HSP70 protein detection. Finally, to assess protein quantity, membranes were stained with either Coomassie brilliant blue or Ponceau S. Images were analyzed in ImageJ as described in [80]. To calculate the changes in HSP70 and HSP90 quantities for each treatment, the respective control values were taken as one (fold change).

### 4.7. Statistical Analysis

In all experiments, at least 3 biological replicates per treatment or control were analyzed. Statistical analysis was performed in the TIBCO Statistica 13.5.0.17 software package (TIBCO Software, Palo Alto, CA, USA). Data were validated with regard to distribution (Shapiro–Wilk test) and variance (Levene’s test) before proceeding with the analysis. One-way ANOVA and post-hoc Tukey’s test (*p* < 0.05) were used to determine the significance. The data were represented as means with standard deviations or box plots. Significant differences are denoted by different letters.

## Data Availability

All row data can be acquired from the corresponding author on request.

## Supplementary Materials

The following supporting information can be downloaded at: https://www.mdpi.com/article/10.3390/ijms24054456/s1.
