# Supplementary material for: Growth and Molecular Responses of Tomato to Prolonged and Short-Term Heat Exposure
Source: Int J Mol Sci. 2023 Feb 24;24(5):4456. doi: 10.3390/ijms24054456 (PMC10002527; doi:10.3390/ijms24054456)
Supplement: Supplementary file 1 [file ijms-24-04456-s001.zip › ijms-2222525-supplementary.pdf]

Supplemental Materials

Growth and Molecular Responses of Tomato to Prolonged and Short-Term Heat Exposure

Table S1. Genes and primer sequences used for quantitative real-time PCR analysis. Reference genes *ACT* and *EFI-α* were used as internal controls for normalization. Genes *DREB1*, *HSFA3*, *HSFB1*, *NAC4* and *NAC6* and appropriate primers were selected based on literature data (last column) and adapted for quantitative PCR analysis.

Gene	Primer	Sequence 5' - 3'	Reference
DREB1 **Solyc06g050520**	qS-DREB1-Fw	CCTAAAAGAGGTAGTAGGTTATG	[77]
	qS-DREB1-Rev	TGCACAAGGACCATACATAGC	
HSFA3 **Solyc09g009100**	qS-HSFA3-Fw	GACAGAAGCAGATGGTTTCATTC	[51]
	qS-HSFA3-Rev	ATTTCCTCATTGTTCTTGGACTAG	
HSFB1 **Solyc02g090820**	qS-HSFB1-Fw	GATGACATAGGTTCAAGTTCTAC	[51]
	qS-HSFB1-Rev	TTCTCATTCTCATCCGACAAGTC	
NAC4 **Solyc11g017470**	qS-NAC4-Fw	CGTCTTATCACGTTTACAATGAC	[48]
	qS-NAC4-Rev	TTCGGCTTCGGCTCACTCT	
NAC6 **Solyc10g055760**	qS-NAC6-Fw	AAGCACGTACACAGCTCTCCT	[49]
	qS-NAC6-Rev	TAACCTATCATCATCTACTTCATCC	
EFI-α **Solyc06g005060**	qS-EFIα-Fw	TACTGGTGGTTTTGAAGCTGG	[76]
	qS-EFIα-Rev	AACTTCCTTCACGATTTCATCATAC	
ACT **Solyc11g005330**	qS-ACT-Fw	TGTCCCTATTTACGAGGGTTATG	[75]
	qS-ACT-Rev	CAGTTAAATCACGACCAGCAAGA	

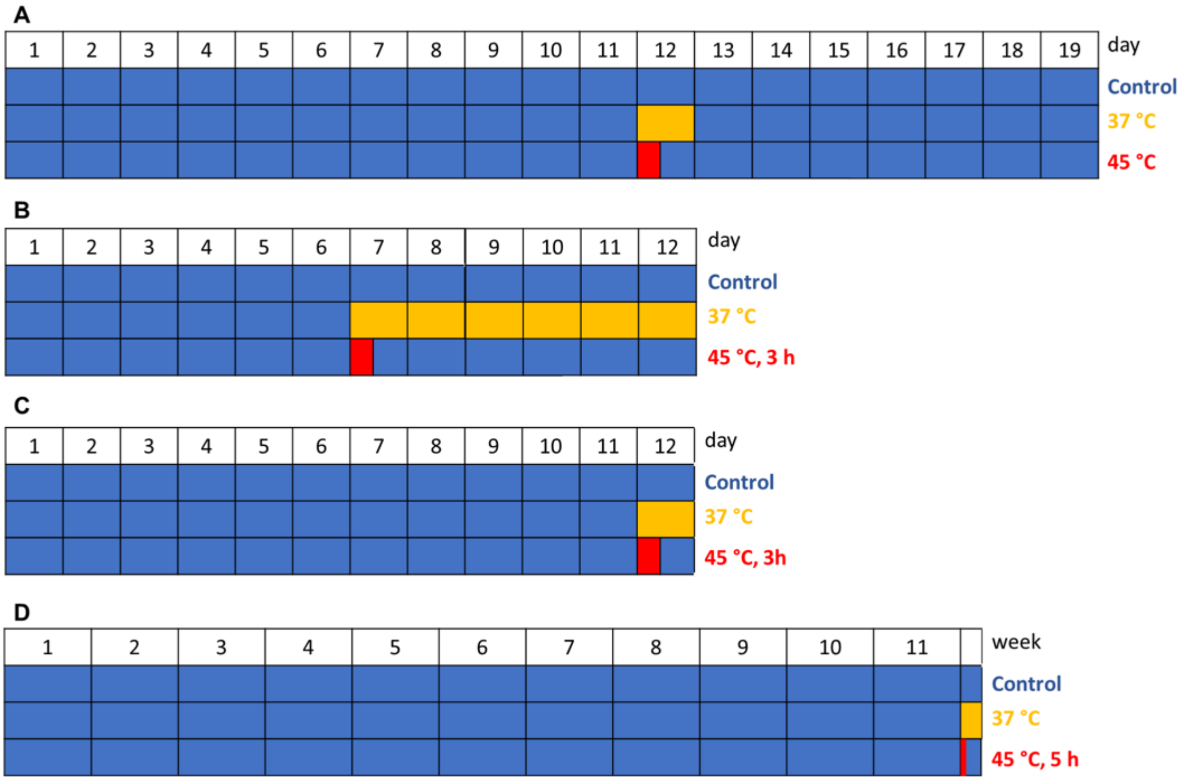

Figure S1. Heat stress treatments of seedlings (A–C) and adult plants (D). Twelve-day old seedlings were exposed to 37 °C for 24 h or to 45 °C for 1, 3, 6 or 12 h and further cultured for 7 days at control conditions (24 °C) when seedling survival rate was determined (A). Seven-day old seedlings were cultured either at 37 °C for 5 days, or exposed to 45 °C for 3 h and further cultured in control conditions at 24 °C (B). Twelve-day old seedlings were exposed to 37 °C for 24 h or to 45 °C for 3 h and further cultured in control conditions at 24 °C (C). Eleven-week old adult plants were exposed to 37 °C for 24 h or to 45 °C for 5 h and further cultured in control conditions at 24 °C (D).
